# Deceptive morphologic and epigenetic heterogeneity in diffuse intrinsic pontine glioma

**DOI:** 10.18632/oncotarget.19726

**Published:** 2017-07-31

**Authors:** Marianna Bugiani, Sophie E.M. Veldhuijzen van Zanten, Viola Caretti, Pepijn Schellen, Eleonora Aronica, David P. Noske, William P. Vandertop, Gertjan J.L. Kaspers, Dannis G. van Vuurden, Pieter Wesseling, Esther Hulleman

**Affiliations:** ^1^ VU University Medical Center, Department of Pathology, Amsterdam, The Netherlands; ^2^ VU University Medical Center, Department of Paediatrics, Division of Oncology/Haematology, Amsterdam, The Netherlands; ^3^ Brain Tumor Center Amsterdam, Cancer Center Amsterdam, VU University Medical Center and Academic Medical Center, Amsterdam, The Netherlands; ^4^ Stanford University School of Medicine, Department of Neurology, Neurosurgery and Pediatrics, Stanford, California, USA; ^5^ Academic Medical Center, Department of Pathology, Amsterdam, The Netherlands; ^6^ VU University Medical Center, Department of Neurosurgery, Amsterdam, The Netherlands; ^7^ VU University Medical Center and Academic Medical Center, Neurosurgical Center Amsterdam, Amsterdam, The Netherlands; ^8^ Prinses Máxima Center for Pediatric Oncology and University Medical Center Utrecht, Utrecht, The Netherlands

**Keywords:** diffuse intrinsic pontine glioma, histone 3, H3 K27M, trimethylation, intratumoral heterogeneity

## Abstract

Historically, the diagnosis of diffuse intrinsic pontine glioma (DIPG) was based on typical imaging findings and clinical characteristics instead of pathology. However, the discovery of mutations in histone H3 variants, and the availability of tumor material for molecular analysis, has led to a paradigm shift in DIPG research and clinical practice. Using data from whole-brain autopsies in a series of nine DIPG patients with known histone mutational status, we here aim to review histopathological characteristics with special focus on intratumoral heterogeneity (ITH) and histone 3 K27 trimethylation (H3 K27me3). All DIPGs showed marked histologic ITH, with 56% even showing focal areas resembling a WHO grade I phenotype. As expected, H3 K27me3 immunoreactivity was lost in the tumors that were H3 K27M-mutated (seven patients; 67% H3.3, 11% H3.1). Strikingly, the H3K27 wildtype tumors (two patients; 22%) also contained H3 K27me3-immunonegative areas. Our study underscores the importance of the choice of the biopsy site, as ITH in DIPGs could theoretically lead to erroneous histological diagnoses with small biopsies. New in this respect is our finding that a substantial number of otherwise typical DIPGs has areas resembling WHO grade I tumors (esp. pilocytic astrocytoma, subependymoma). Furthermore, our study shows that negative H3 K27me3 immunohistochemistry in a DIPG does not imply a H3 K27-mutant tumor.

## INTRODUCTION

Diffuse intrinsic pontine glioma (DIPG), one of the deadliest childhood cancers [1], shows marked intratumoral heterogeneity (ITH) in terms of histological phenotype and malignancy grade [2]. Because of this heterogeneity and the possible risks associated with biopsy procedures, DIPG diagnoses have been made solely on clinical and radiological grounds for decades. Owing to the reintroduction of stereotactic and open surgical biopsies and subsequent molecular characterization of these tumors, it was recently demonstrated that over 90% of DIPGs carry a histone H3 K27M mutation in the genes encoding H3.3 (*H3F3A*), H3.2 (*HIST2H3C*), or H3.1 (*HIST1H3B* and *HIST1H3C*). Moreover, it was shown that these mutations are spatially conserved and result in both loss of H3 K27 trimethylation (H3 K27me3) and alteration of gene expression profiles, putatively driving gliomagenesis [3]. Since H3 K27 mutations are significantly associated with survival, the *2016* World Health Organization (WHO) Classification of Tumours of the Central Nervous System introduced “Diffuse midline glioma, H3 K27M-mutant” as a separate WHO grade IV entity, wherein DIPG is a relatively frequent subgroup [4]. Consequently, immunohistochemistry for H3 K27M-mutant protein and for H3 K27me3 is now increasingly used to assess the H3 K27 status of DIPGs. Based on these recent discoveries, this study aims to perform integrated morphologic and molecular characterization in a series of DIPG patients in whom whole-brain autopsies were performed. Autopsies were performed according to an established protocol [5] that was approved by the medical ethical committee of the VU University Medical Centre, Amsterdam, The Netherlands.

## RESULTS

### Patient characteristics and autopsy procedure

All patients fulfilled the diagnostic MRI criteria for DIPG according to Barkovich et al [6], i.e. a T1-weighted hypointense and T2-weighted hyperintense tumor with at least 50% involvement of the pons. Table 1 shows the patient and treatment characteristics. Median age at diagnosis was 9.0 years (range 1.3-14.7 years). In most patients, symptoms preceded presentation by less than 6 weeks. In two patients, symptom duration was longer than three but less than six months. The complaints in the latter cases included gait disturbances and strabismus.

**Table 1:** Clinical characteristics of DIPG patients

Patient ID	Age (y)	Gender	Symptom duration (w)	Treatment at diagnosis	Treatment at disease progression	PFS (m)	OS (m)
1	5.0	M	<6	XRT (15×3=45 Gy)	-	8	8
2	9.0	F	12-24	XRT (16×2.8 = 44.8 Gy)	Temozolomide	28	41
3	1.3	F	12-24	-	Vincristine, carboplatin	2	11
4	7.5	M	<6	XRT (13×3.0=39 Gy) + temozolomide	-	5	6
5	7.2	M	<6	XRT (30×1.8=54Gy) + gemcitabine / HDC / combined targeted therapy	Temozolomide, imatinib, dichloroacetate	12	20
6	14.7	M	<6	XRT (6×3.0=18 Gy)	-	1	6
7	11.1	M	<6	XRT (13×3.0=39 Gy)	XRT (8×3.0=24 Gy)	12	17
8	10.5	M	<6	XRT (13×3.0=39 Gy)	XRT (10×3.0=30 Gy)	2	15
9	12.3	F	<6	XRT (30×1.8=54Gy) + gemcitabine	-	1	4

M: male, F: female, y: years, m: months, w: weeks, h: hours, XRT: radiotherapy, HDC: high-dose chemotherapy, PFS: progression-free survival, OS: overall survival

All patients except the youngest one received radiotherapy at time of diagnosis (range 18-54 Gy; Table 1). The median progression-free survival was 5 months (range 1-28 months). At time of progression, three patients received chemotherapy and two underwent re-irradiation. Median overall survival was 11 months (range 4-41 months).

Autopsies were performed within 7 hours after death (mean post-mortem time 3 hours). Per autopsy, an average total of 7 tumor samples from the pons, cerebellar peduncles, and cerebellar hemispheres were obtained. Additionally, an average of 27 samples of histopathologically normal tissue were obtained from adjacent areas cranially and caudally within the brainstem and from more distant cerebral regions, resulting in a total of 306 study samples across all patients.

### Immunohistochemistry and molecular analysis

**Figure 1:**
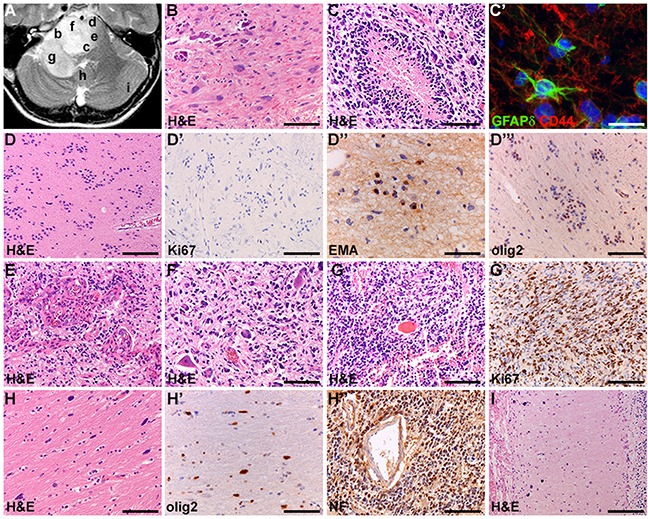
Intratumoral heterogeneity of DIPG (patient VUMC-DIPG-6) **(A)** axial T2-weighted MR-image; letters correspond to tumor areas illustrated. **(B)** Gemistocitic morphology of tumor cells and calcifications. **(C)** High-grade component with necrosis surrounded by pseudopalissading of tumor cells. **(C')** In the same area, tumor cells expressing the stem cell markers GFAPδ and CD44. **(D)** Subependymoma-like component showing negligible mitotic activity **(D')**, dot-like cytoplasmic immunopositivity for the epithelial membrane antigen (EMA, **D''**), indicating ependymal differentiation, and immunopositivity for olig2 **(D''')**. **(E)** High-grade component with microvascular proliferation. **(F)** Tumor cells infiltrating the gray matter of the pontine nuclei. **(G)** High-grade component with small cell morphology and brisk mitotic activity **(G')**. **(H)** Low-grade component with isolated tumor cells infiltrating the distant white matter and expressing olig2 **(H')**. In the same area, perivascular clustering of tumor cells immunopositive for neurofilament (NF) 70-200kDA **(H'')**. **(I)** Isolated tumor cells in the distant cerebellar cortex. Bars: 100μm, C' 200μm.

All DIPGs showed marked histologic ITH including areas with WHO grade II–IV histology. Additionally, focal areas were present with a pilocytic astrocytoma- and/or subependymoma-like phenotype (3 and 5 cases, respectively), thus resembling WHO grade I lesions (Figure 1). Pilocytic astrocytoma-like regions were characterized by piloid tumor cell processes and presence of Rosenthal fibers, while subependymoma-like areas were paucicellular with marked clustering of tumor cells, some of which showing dot-like EMA-positivity, and some expressing the glial-restricted progenitor cell marker Olig2. Immunohistochemistry confirmed the substantial ITH with respect to astrocytic differentiation and expression of stem cell markers (GFAP, GFAPδ, nestin and CD44), only partly overlapping with the degree of cellular atypia, and revealed at least focal expression of neuronal markers (including synaptophysin and neurofilament 70-200kDa).

**Figure 2:**
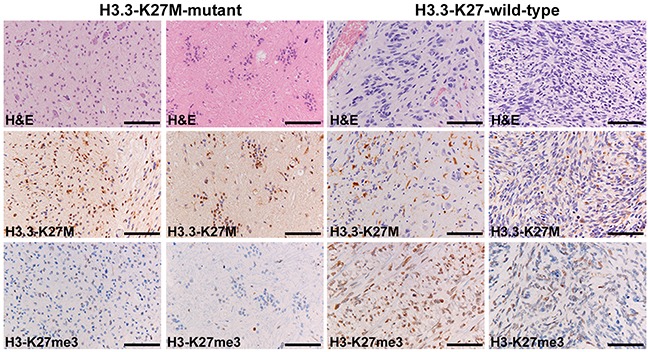
Immunohistochemistry for the H3 K27M-mutant protein and H3 K27me3 Patient VUMC-DIPG-6 (H3. 3 K27M-mutant, left panels) is immunopositive for the mutant protein in tumor nuclei and shows H3 K27me3-loss in both high-grade (left) and low-grade (middle-left) components. Patient VUMC-DIPG-1 (H3 K27 wildtype, right panels) is immunonegative for the mutant protein in tumor cells, with aspecific microglia staining. Stain against H3 K27me3 shows H3 K27me3-conservation in some areas (middle-right), whereas other areas display H3 K27me3-loss (right). Bars: 100μm.

Table 2 shows the results of the whole-genome paired-end sequencing and Sanger sequencing. Six tumors (67%) carried a H3.3 K27M mutation, one (11%) carried a H3.1 K27M mutation, and two tumors were H3 K27 wildtype (22%). All H3.3 K27M and H3.1 K27M tumors were immunopositive for the mutant protein. Strikingly, the H3 K27 wildtype tumors (two patients; 22%) also contained H3 K27me3-immunonegative areas (Figure 2).

**Table 2:** Whole-genome paired-end and Sanger sequencing results

Patient ID	H3.3 K27M	H3.3 G34R/V	H3.1 K27M
1	WT	WT	WT
2	WT	WT	mut
3	mut	WT	WT
4	mut	WT	WT
5	mut	WT	WT
6	mut	WT	WT
7	mut	WT	WT
8	mut	WT	WT
9	WT	WT	WT

WT: wildtype, mut: mutant

## DISCUSSION

Based on our findings in an autopsy series of nine DIPG patients evaluated in the last eight years at the VU University Medical Center Amsterdam, we underscore that DIPG ITH may lead to misinterpretation in biopsy specimens at two distinct levels when using (immuno) histochemistry only:

*1. Histologic phenotype*. Without exception, all DIPGs showed marked histologic ITH. Remarkably, 56% also showed focal areas with a pilocytic astrocytoma- and/or subependymoma-like phenotype resembling WHO grade I lesions. This finding is of interest, since many consider WHO grade I lesions not part of the histologic spectrum of DIPG, even in case of typical imaging features. Patients with a WHO grade I lesion upon biopsy were therefore often excluded from clinical DIPG trials. With our results we show that this may have potentially been a “false negative” decision. Of note, the survival of the patients with WHO grade I-like tumor regions was as poor as that of the others.

*2. Immunohistochemical assessment of H3 K27me3/H3 K27 status*. As expected, all H3 K27M tumors (78%) lost H3 K27me3 immunoreactivity, irrespective of histological phenotype and grade. However, the two H3 K27 wildtype neoplasms also contained H3 K27me3-immunonegative areas, which were not clearly related to tumor morphology or grade. Using only H3 K27me3 immunohistochemistry may thus lead to a false positive diagnosis of H3 K27-mutant glioma. Focal loss of H3 K27me3 has been reported only once in 2 out of 76 high-grade gliomas [7] and may indicate additional mechanisms underlying the previously described dominant-negative effect of the H3 K27-mutant protein on H3 K27me3. Further research investigating these mechanisms may help to better understand the H3 K27 wildtype subgroup, with possible diagnostic and therapeutic consequences. If loss of H3 K27me3 actively contributes to a more malignant course of the disease, H3 K27me3 immunodetection could add important information. Multiple biopsies for multiregional H3 K27me3 immunodetection should then be considered, especially when therapies targeting loss of H3 K27me3 could also be partly effective in patients with H3 K27 wildtype tumors. Notably, interpretation of immunohistochemical staining for the H3 K27-mutant protein may be challenging, especially as discriminating positive microglial cells from positive tumor cell nuclei can be difficult. Ideally, molecular analysis should be performed for a definitive assessment the H3 K27 status of the tumor.

In conclusion, here we demonstrate that histologic phenotype and immunohistochemical staining for H3 K27 status in small DIPG biopsies can be deceptive. From a diagnostic point of view this means that to make the diagnosis DIPG, typical imaging- and clinical signs remain crucial. And, to make the diagnosis of the (overarching) entity diffuse midline glioma, H3 K27M-mutant, as defined in the WHO 2016 classification, next generation sequencing/Sanger sequencing for now seems more reliable than H3 K27M/H3 K27me3 immunohistochemistry. Future studies are needed to assess the correlation between (heterogeneous) focal loss of H3 K27me3 in H3 K27-wildtype DIPGs and the clinical behavior of these tumors. Last but not least, from a therapeutic point of view, the observed spatial heterogeneity in DIPGs suggests that future chemotherapy should be directed against intratumoral polyclonality using multiple drugs, instead of therapies targeting only a single (putative) target. Moreover, combining this with various administration modalities (i.e. systemic and local drug delivery, such as convection enhanced delivery or ultrasound mediated blood-brain barrier disruption) and (re)irradiation schedules may help to better target the full range of this heterogeneous disease.

## METHODS

Whole-brain autopsy was performed in nine DIPG patients diagnosed based on clinical and MRI features [6] according to the ethical approved protocol [5]. The brainstem and cerebellum were separated from the cerebral hemispheres and cut axially. The cerebral hemispheres were cut coronally. Tumor location and extension via direct parenchymal infiltration and leptomeningeal or intraventricular invasion was evaluated macroscopically. Multiple tissue samples were collected from the pons, cerebellar peduncles, cerebellar hemispheres, medulla oblongata, cervical spinal cord, midbrain, thalami, wall of the third ventricle, subventricular zone, basal nuclei, hippocampi, and all cerebral lobes. Both tumorous and histopathologically normal brain tissue were collected.

Four-μm-thick sections of formalin-fixed paraffin- embedded material were histochemically stained for Hematoxylin & Eosin (H&E). After heat-induced antigen retrieval in 0.01M citrate buffer (pH6), immunohistochemical staining was performed with antibodies against glial fibrillary acidic protein (GFAP; Sigma, G3893, 1:1000), neurofilaments 70-200 kDa (Monosan, 1:10), oligodendrocyte transcription factor 2 (olig2; Abcam, ab33427, 1:400), Ki67 (Dako, M7240, 1:160), epithelial membrane antigen (EMA; Dako, M0613, 1:100), H3.3 K27M (Merck Millipore, ABE419, 1:500) and H3 K27me3 (Abcam, ab24684, 1:100). Immunopositivity was detected with 3,3′-Diaminobenzidine (DAB) as chromogen. Six-μm-thick frozen tissue sections were used for fluorescence immunohistochemistry. Tissue sections were fixed in 4% paraformaldehyde, blocked for 30 minutes in phosphate buffered saline supplemented with 0.1% saponin and 5% normal goat serum, and incubated with antibodies isoform GFAP delta (GFAPδ; kind gift of E. Hol, University Medical Centre Utrecht, 1:250 [8]) and CD44 (Hermes3, 1:100 [8]). After incubating with secondary antibodies (Alexa Fluor 488- or 594-tagged; Molecular Probes, 1:400), sections were counterstained with 4′,6-diamidino-2-phenylindole (DAPI; Molecular Probes, 10 ng/ml) and embedded in Fluoromount G (Southern Biotech). All tissue sections were photographed using a Leica DM6000B microscope (Leica Microsystems). Omitting primary antibodies yielded no significant staining.

All nine tumors were molecularly characterized by next generation sequencing, and histone variants were validated with Sanger sequencing. Genomic DNA was isolated from DIPG tumors and from non-affected counterpart brain regionsusing the QIAamp DNA mini isolation kit (Qiagen, USA). Next generation sequencing was executed by whole-genome paired-end sequencing as performed by Complete Genomics [9]. Primary data analysis – including variant calling – was performed and mapped to reference build hg19. Genomic data was further analyzed using the open source CGAtools (http://cgatools.sourceforge.net), generating somatic calls for splice sites or coding exons. For Sanger sequencing, the histone 3 sequences of interest were amplified by PCR andsubsequently sequenced by the dideoxy chain-termination method using the ABI PrismTM BigDye Terminator kit (Perkin Elmer, USA), which was run on the ABI Prism Genetic Analyser 3100 automatic DNA autosequencer (Perkin Elmer) and analysed with ABI sequence Alignment Editor software. Primers were designed using Oligo explorer 1.5 software (Genelink – primer sequences available upon request) and blasted against the human genome for specificity (NCBI).

## Abbreviations

DAB: 3,3′-diaminobenzidine
DIPG: diffuse intrinsic pontine glioma
EMA: epithelial membrane antigen
GFAP: glial fibrillary acidic protein
GFAPδ: isoform delta of GFAP
HIST2H3C: gene encoding H3.2
HIST1H3B: gene encoding H3.1
HIST1H3C: gene encoding H3.1
H3F3A: gene encoding H3.3
H3 K27me3: H3 K27 trimethylation
H&E: hematoxylin & eosin
ITH: intratumoral heterogeneity
olig2: oligodendrocyte transcription factor 2
WHO: World Health Organization
